# Induction of resistance of *Podosphaera xanthii* (hull-less pumpkin powdery mildew) to triazole fungicides and its resistance mechanism

**DOI:** 10.1371/journal.pone.0263068

**Published:** 2022-02-01

**Authors:** Qiaolan Liang, Liexin Wei, Bingliang Xu, Jia Liu, Shuwu Zhang, Lilong Liu

**Affiliations:** 1 College of Plant Protection of Gansu Agricultural University; Lanzhou, P.R. China; 2 Biocontrol Engineering Laboratory of Crop Diseases and Pests of Gansu Province, Lanzhou, P.R. China; Bangabandhu Sheikh Mujibur Rahman Agricultural University, BANGLADESH

## Abstract

The aim of this study was to uncover the molecular mechanism through which fungicide resistance develops in *Podosphaera xanthii*, a fungi that causes powdery mildew in hull-less pumpkin. Treatments of inoculated *P*. *xanthii* were carried out on leaves of hull-less pumpkin and subsequently treated with kinds of triazole fungicide for seven generations. Resistant strains of *P*. *xanthii* thus obtained were evaluated for their resistance levels. The resistance levels of the fungi to four fungicides of were high except that of the propiconazole-resistant strain, which showed moderate resistance. The F7 generations of five resistant strains thus obtained were cultured continuously for five generations without fungicide induction, and their resistance level were found to be relatively stable. The DNA of the sensitive strain and the five kinds of resistant strains were extracted by the sodium dodecyl sulfate (SDS) method and its internal transcribed spacer (ITS) region was amplified by using ITS1/ITS4 primer and specific primer F/R and they were sequenced respectively. The DNA sequence comparison of resistant and sensitive strains showed that the base pairs of tebuconazole-resistant strains and flusilazole-resistant strains were mutated, with mutation rates of 4.8% and 1.6%, respectively. The base pairs of the other three resistant strains did not change.

## Introduction

Hull-less *Cucurbita pepo* is an annual herb of the family Cucurbitaceae. As its seeds have seed kernels and no seed coat, it is also called hull-less pumpkin, which is a rare variant of *Cucurbita*. Hull-less *C*. *pepo* has high nutritional and utilization value, as it not only contains nutrients such as vitamins, amino acids and unsaturated fatty acids necessary for the human body, but it also has therapeutic uses, especially in the prevention and adjuvant treatment of diabetes, as well as being valued as a raw material for cosmetics, health care products and oils [1]. Previous studies have shown that hull-less pumpkin seed oil press-cake flour, as a by-product and functional ingredient, has also been incorporated into gluten-free cookies with the aim of improving their nutritional quality [1].

The extract of hull-less pumpkin seed (HLPS) has significant anti-cancer effects [2]. The total oil, total phenol content, and antioxidant activity values of seed oils were found to be between 33.04 and 46.97%, 56.94 and 87.15 mg GAE/100 g, 0.19 and 11.75%, respectively (p < 0.05). Linoleic, oleic, palmitic and stearic acids were the most prominent fatty acids in all genotypes. The most abundant mineral in the studied seeds, which belong to different genotypes, was potassium (2704.75–1033.63 ppm) followed by phosphorus (3569.69–9108.84 ppm) and magnesium (1275.15–3938.16 ppm) (*p* < 0.05) [3]. In Poland, Styrian hull-less pumpkins are valued for their use in health-promoting foods such as oils and snacks, and although indigenous to Styria, are now cultivated globally [4]. In Japan, a new pumpkin cultivar ’Stripe *pepo*’ with hull-less seeds and short internodes was bred and the fruit rind is orange with green stripes; shows a higher seed yield [5]. In recent years, the hull-less pumpkin has shown remarkable economic benefits [6]. Presently, the hull-less pumpkin is cultivated in eleven provinces of China, and the output is approximately 117 million tons, accounting for 1/3 of the total output worldwide [7,8]. It has played an important role in increasing farmers’ income and export earning foreign exchange to promote local economic development.

However, with the continuous expansion of the cultivation area of the hull-less pumpkin, the occurrence of powdery mildew (*P*. *xanthii*) has become increasingly serious, which has become the main constraining factor for pumpkin production in China [9,10]. Cucurbit powdery mildew is a serious disease that impacts field and greenhouse cucurbit crops worldwide, which is caused most frequently by two kinds of obligate parasite of (*Golovinomyces (Erysiphe) orontii* s.l., and *P*. *xanthii*) [11–13]. The pathogen causing hull-less pumpkin (*C*. *pepo*) powdery mildew in the Western District in China was identified as *P*. *xanthii* (MT250855) based on the morphology of cleistothecia and conidia, conidial germination, host range and areas where disease occurs, etc. [10,13]. Pumpkin powdery mildew can cause slow growth of plants, premature desiccation of the leaves, and consequent reduction of the quality and marketability of the fruit [14]. Presently, the pumpkin powdery mildew is mainly controlled by using disease-resistant varieties and chemical fungicides, but the acquisition of disease-resistant germplasm is limited by time, manpower, and financial resources. Fungicides have the characteristics of rapid control and quick effect in plant disease control; therefore, chemical control is still the main control measure of pumpkin powdery mildew.

Among many fungicides, triazole fungicides are highly efficient agents used to control cucurbit powdery mildew in agricultural production in China. However, the rapid development of pathogens resistant to fungicide has now become a prominent problem that limits the effectiveness of fungicides. In recent years, there have been increasingly frequent reports on the use of molecular methods to study the resistance of pathogens to fungicides. Studies have shown that the main cause of resistance of pathogens to 14ɑ-demethylation inhibitors (DMIs) are point mutations in the *CYP51* gene [15]. In France, Délye (1997) found that the pathogen caused wheat powdery mildew is resistant to triazole fungicides because of mutations in the *CYP51* gene alleles [16]. Further studies by Délye revealed that resistance to triadimefon in barley powdery mildew, and grape powdery mildew, are caused by *CYP51* gene mutations resulting in tyrosine at position 136 to be replaced by phenylalanine [17]. In China, the resistance of isolates to triadimefon was caused not only by the single-site mutation in the gene, which was found by detecting the resistant site 136 in the *CYP51* gene of wheat powdery mildew population with the technology of allele-specific Polymerase Chain Reaction (AS-PCR), as well as cloning and sequencing. Comparing the sequence of the resistant and the sensitive isolates, it was found there were another five mutation sites in the 175^th^, 185^th^, 398^th^, 419^th^ and 430^th^ sites of the amino acids [18]. Presently, molecular methods have not been used to study the resistance of hull-less pumpkin powdery mildew to triazole fungicides. In this experiment, the resistance of sensitive strain of hull-less pumpkin powdery mildew was induced by using five kinds of triazole fungicides in laboratory in order to better understand the resistance generation and determine the resistance stability. The DNA of resistant and sensitive strains were extracted, amplified and sequenced after seven consecutive generations of fungicide treatment by the same method and F7 resistant strains after continuous culture for five generations. The resistance mechanism was clarified by comparing the DNA sequence of resistance strains with that of sensitive strains.

## Materials and methods

### The pumpkin varieties and the strains of pumpkin powdery mildew

Seeds of “Tianran”, a variety of hull-less pumpkin sensitive to *P*. *xanthii* were provided by Wuwei Golden Apple Co., Ltd. of Gansu Province and planted in an artificial intelligence climate box. These pumpkin seedlings were used to propagate sensitive strains of pumpkin powdery mildew (denoted by the abbreviation SY2) [19] and as a host for the later tests. Sensitive strain of pumpkin powdery mildew (SY2) was preserved in hull-less pumpkin seedlings (two leaf stage) planted in the nutrient bowl and placed in the laboratory.

Test triazole fungicides are listed in Table 1.

**Table 1:** Test fungicides.

Fungicide	Manufacturer	Recommended concentration μg·mL^-1^
2.5% propiconazole EC	ShaanxiBailu Agrochemical Co., Ltd.	1066
10% flusilazole EW	Qingdao Qinsheng Biotechnology Co., Ltd.	2000
5% hexaconazole ME	Qingdao Dongsheng Pharmaceutical Co., Ltd.	1667
12.5% myclobutanil EC	Huabei Pharmaceutical Group Ainuo Co., Ltd.	1667
25% tebuconazole EC	Shanghai Aix Biopharmaceutical Co., Ltd.	2766

### Main reagent and preparation method

Main reagent: SDS; Tris; EDTA; Chloroform; Isopentanol; Ammonium acetate; Sodium acetate; Anhydrous ethanol; Isopropyl alcohol; glacial acetic acid; agarose; PCR master mix; deoxyribonucleosides Triphosphoric acid; RNase enzyme, etc. The following reagent solutions were prepared according to the test requirements.

1 mol·L^-1^ Tris-HCl; 0.5 mol·L^-1^ EDTA; DNA extraction buffer (pH 8.0); 7.5 mol·L^-1^ NH_4_AC; 3 mol·L^-1^ NaAC (pH 5.2) TE buffer solution; 20 mg·mL^-1^ RNase enzyme (sub- packaged and stored at temperature of -20°C for standby, avoid repeated freezing and thawing); Chloroform isoamyl alcohol solution (24:1 ratio by volume of chloroform to isoamyl alcohol); 70% ethanol; 0.5 μg·mL^-1^ ethidium bromide; etc.

### Main instruments and equipment

High speed freezing centrifuge (Jining Yuze Industrial Technology Co. LTD, Xiangyi H1650); Agarose gel electrophoresis system (Beijing Liuyi Biological Co., LTD., DYY-6D); PCR gene amplification instrument (BIO RAD, T100); UV Gel imaging analysis system (ESSENTIAL, V6); Vortex oscillator (scilogex, MX-S); Digital thermostat water bath (Shanghai Yuejin Medical Device Co., LTD., HSY-12); Ultrapure water preparation instrument (Chengdu Kangshi Corning Technology Development Co., LTD.); Clean bench (Haier, HCB-1300V); artificial intelligence climate box (Hangzhou Qisheng Electronic Technology Co., LTD, QRGN—400–3); Autoclave (Shanghai Shen ’an, LDZF-75L-11).

### Obtaining triazole fungicide resistant strain of powdery mildew

The conidia of the diseased leaves with SY2 strain inoculation healthy hull-less pumpkin seedlings (two leaf stage) were brushed, and the residue was used to prepare conidia suspension, spore suspension of sensitive strain SY2 of concentration 7–8×10^5^ conidia·mL^-1^ was prepared with sterilized water. This conidia suspension was inoculated on healthy pumpkin leaves by the daubing method, placed in an artificial intelligence climate box at 25 °C, relative humidity (RH) 60%, 4400 lx light for 12 h/d and cultured for 3 days.

The recommended concentration of each fungicide in Table 1 was taken as the highest concentration and diluted into five serial concentrations by using the half-dilution method.

Five small sprayers were used to uniformly spray the above serial concentrations of triazole fungicides on the pumpkin seedlings with sensitive strains inoculation after cultured for 3 days. Ten seedlings were sprayed per treatment, with three repetitions each. Additionally, seedlings sprayed with sterilized water were used as a control. These were dried, and placed in an artificial intelligence climate box at 25 °C, relative humidity (RH) 60%, 4400 lx light for 12 h/d and cultured for a further seven days. The diseased leaves were examined according to the following grading standards (Table 2), the incidence, disease index and control effect were calculated according to formula (1), (2) and (3). Inoculation of leaves with 100% incidence is the premise to ensure the development of the experiment. SPSS 20.0 software was used to calculate the linear regression of the concentration logarithm, control effect probability value, simulate the regression equation, and calculate the EC_50_ value of each fungicide to the sensitive strain [10,11].


$$incidence=\frac{\;inoculation\;diseased\;leaves}{inoculation\;total\;number\;of\;leaves}\times 100\%$$ (1)

$$disease\;index=\frac{\sum \left(diseased\;leaves\times graded\;number\right)}{survey\;total\;number\;of\;leaves\times total\;graded\;number\;}$$ (2)

$$control\;effect=\frac{control\;disease\;index-treatment\;disease\;index}{control\;disease\;index}\times 100\%$$ (3)

**Table 2:** Graded standards for pumpkin powdery mildew.

Graded level	Disease spot area	Graded level	Disease spot area
Level 0	Without disease spot	Level 3	1/3≤Diseased spots occupy leaf area<1/2
Level 1	Diseased spots occupy leaf area<1/5	Level 4	1/2≤Diseased spots occupy leaf area<2/3
Level 2	1/5≤Diseased spots occupy leaf area<1/3	Level 5	Diseased spots occupy leaf area≥2/3

The conidia of the diseased leaves with SY2 strain inoculation were brushed, and the residue was used to prepare conidia suspension, which was then reinoculated on healthy pumpkin leaves. After seven days of culturing in an artificial intelligence climate box under the same conditions as mentioned previously, the triazole fungicides water dilution concentration was prepared according to EC_50_ value sprayed on the leaves of seedlings that were inoculated. The powdery mildew conidia obtained from the diseased leaves were used as the first generation of the triazole fungicide induced screening, and the EC_50_ and resistance levels of each generation were determined after seven consecutive generations by the same method. The EC_50_ of F7 resistant strains after continuous culture for five generations (F8, F9, F10, F11, F12) and resistance levels of each generation to each of the five kinds of triazole-fungicide-resistant induced strains of pumpkin powdery mildew were determined respectively.

The resistance factor of the first, third, fifth, and seventh generations were calculated according to formula (4), and the resistance level evaluation standards are shown in Table 3.


$$Resistance\;factor\left(RF\right)=\frac{EC_{50}\;of\;resistance\;strain}{EC_{50}\;of\;sensitive\;strain}$$ (4)

**Table 3:** Evaluation standards of pumpkin powdery mildew resistance level.

Resistance factor (RF)	Resistance level	Resistance factor (RF)	Resistance level
<3.0	sensitive	>10.0	obvious resistance
3.1~5.0	resistance	10.1~40.0	moderate resistance
>5.0	mild resistance	40.1~160.0	highly resistance
5.1~10.0	low resistance	>160.0	extremely highly resistance

### Molecular mechanism of resistance to triazole fungicides in hull-less pumpkin powdery mildew

#### Collection of conidia of sensitive strain and resistant strains of hull-less pumpkin powdery mildew

The conidia of the pumpkin powdery mildew sensitive strain and resistant strains were made into a suspension (concentration of 7–8×10^5^ conidia·mL^-1^), inoculated by daub method on the leaves of hull-less pumpkins grown in the laboratory, and placed in an artificial intelligence climate box of 25 °C, RH 60%, 4,400 lx light for 12 h/12 h (L/d). After 14 days of cultivation, powdery mildew spores were collected on a clean bench when a large number of conidia formed on the leaves. Cut-off leaves of the diseased hull-less pumpkin were placed on cellophane and gently tapped leave twice to thrice with tweezers to shake off a large number of conidia, the remaining conidia on the leaves were then removed with a fine brush, finally, all the collected powdery mildew spores were collected into a 2 mL centrifuge tube, not amounting to more than 50 to 100 mg of powdery mildew spores in each tube. After each collection in the clean bench was finished, the clean bench was wiped with 70% alcohol for sterilization, then the UV lamp and blower were turned on for 10 min in order to prevent cross contamination between strains. The centrifuge tube containing the spores was placed in a desiccator, dried for 3 to 5 days, and then stored in a refrigerator at -20 °C.

#### Extraction of total DNA of hull-less pumpkin powdery mildew and its quality detection

The total DNA content of hull-less pumpkin powdery mildew sensitive strain and resistance strains were extracted by the summarized and modified sodium dodecyl sulfate (SDS) method according to Liang QL’ research group [20,21].

Detection of extracted DNA were carried out through 1.0% agarose gel electrophoresis using marker (DNA Marker D, Shanghai Sheng gong), 0.5 μg·mL^-1^ ethidium bromide staining for 30 minutes, observing and photographing in UV Gel imaging analysis system and its concentration was by DNA concentration meter.

#### Primer design and PCR amplification

In this experiment, the universal primers ITS1 (5’-TCC GTA GGT GAA CCT GCG G-3’) and ITS4 (5’-TCC TCC GCT TAT TGA TAT GC-3’) synthesized by Shanghai Bioengineering Co., Ltd were selected to amplify the sensitive and resistant strains.

The PCR reaction was carried out using 25 μL Dream Taq Green PCR Master Mix (2×), 1 μL ITS1, 1 μL ITS4, 1 μL Template DNA sequentially added in a 0.5 mL PCR tube, made up to a volume of 50 μL with Nuclease-Free Water.

PCR amplification were carried out according to the cycling conditions detailed in Table 4, and the PCR products were subjected to 1.0% agarose gel electrophoresis to detect the PCR results.

**Table 4:** PCR thermal cycling conditions.

Step	Temperature (°C)	Time	Number of cycles
Initial denaturation	95	3 min	1
Denaturation	95	30 s	38
Annealing	55	30 s	
Automated fluorescent extension	72	1 min	
Final extension	72	10 min	1

According to the resistance of wheat powdery mildew to triadimefon, the *CYP51* gene in the wheat powdery mildew colony has a mutation site in amino acid 398 positions in the wheat powdery mildew population, and the specific primers are designed as follows:

F: 5’-TCA TAC AGA GCA CCA AGA ACA TTA-3’
R: 5’-GTA CAA TAG CAG GTG GAG TT-3’

Five kinds of resistant strains DNA were amplified by PCR which using F, R primer.

### Sequence determination and comparison

The results of PCR amplification of the five resistant strains and one sensitive strain were sent to Shanghai Biotech Co., Ltd. for bidirectional sequencing, each strain was repeated thrice. The sequence alignment of each strain was performed, the base pair of the variation and mutation rates were calculated after compared the sequences of the five resistant strains with the sequences of the sensitive strain by DNAman software V6.

## Results

### Obtaining resistant strains of powdery mildew

Five kinds of resistant strains with different levels of resistance were obtained after seven *P*. *xanthii* generations by using five kinds of triazole fungicides. The results showed that the resistance of the powdery mildew strains increases with an increase in the number of inductions. The resistance factor of *P*. *xanthii* induced by 10% fluorosilazole EW, 5% hexaconazole ME, and 12.5% myclobutanil EC lay between the resistances induced by the propiconazole EC and tebuconazole EC, at 56.71, 49.04, 47.89, respectively (Table 5). Additionally, the resistance level of the other four resistant strains reached high resistance levels but the resistance level of the propiconazole-resistant strain remained at moderate levels.

**Table 5:** Determination of resistance of pumpkin powdery mildew induced by five triazole fungicides.

Fungicides	F1		F3		F5		F7	
	RF	Resistance level	RF	Resistance level	RF	Resistance level	RF	Resistance level
25% propiconazole EC	4.94	resistance	6.81	low resistance	9.23	low resistance	19.11	moderate resistance
10% flusilazole EW	5.57	low resistance	9.97	low resistance	46.47	highly resistant	56.71	highly resistance
5% hexaconazole ME	4.92	resistance	5.02	low resistance	22.56	moderate resistance	49.04	highly resistance
12.50% myclobutanil EC	3.36	sensitive	5.57	low resistance	15.21	moderate resistance	47.89	highly resistance
25% tebuconazole EC	3.22	sensitive	6.00	low resistance	34.13	moderate resistance	64.62	highly resistance

**Fig 1:**
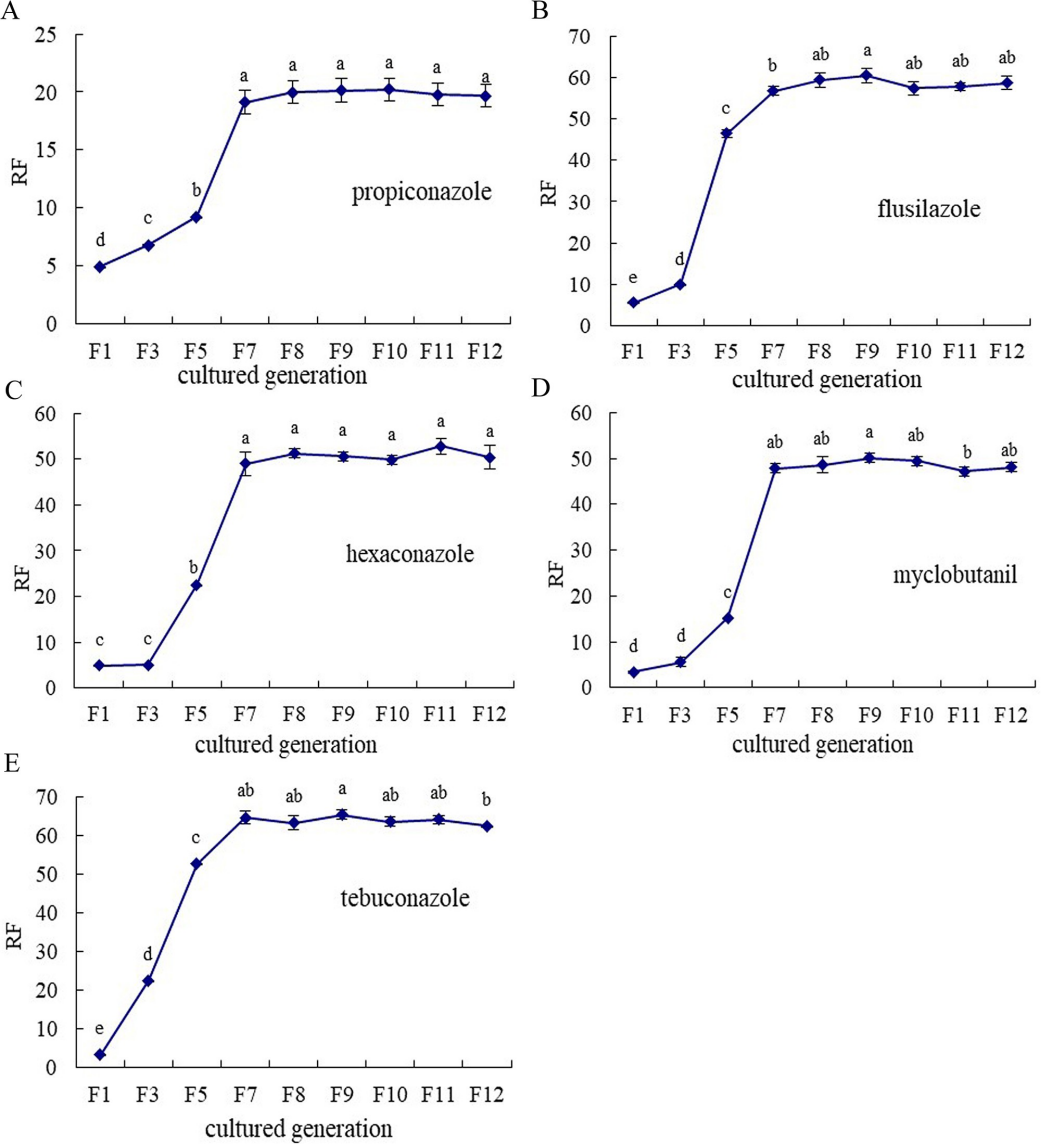
Resistance stability of five kinds of triazole-fungicides-resistant strains to pumpkin powdery mildew. A: Resistance stability in strains resistant to propiconazole. B: Resistance stability in strains resistant to flusilazole. C: Resistance stability in strains resistant to hexaconazole. D: Resistance stability in strains resistant to myclobutanil. E: Resistance stability in strains resistant to tebuconazole. Lowercase letters indicates significant difference in 0.01 level.

The virulence of five kinds of F7 resistant strains were assayed after continuous culture for five generations in non-fungicide pumpkin seedlings (F8, F9, F10, F11, F12) and their resistance factor were calculated. The results indicated that the resistance levels of the five resistant strains showed a wave-like pattern with little change and there were no significant differences between the resistance factors of F8, F9, F10, F11, F12 generation and F7 generation except for flusilazole and tebuconazole (Fig 1B and 1E; p<0.05). It was shown that the resistance of resistant strains of propiconazole, hexaconazole and myclobutanil remained stable (Fig 1A, 1C and 1D; p<0.05).

### Molecular mechanism of the hull-less pumpkin powdery mildew of resistance to triazole fungicides

#### Extraction of total DNA from five kinds of resistant strains and one sensitive strain

The purity and concentration of the extracted DNA of five resistant strains and one sensitive strain meet the reaction requirements of the PCR, which could be judged by the brightness of the strip, and the concentration of extracted DNA was more than 200 ng/μL by DNA concentration meter determined.

#### PCR amplification

The extracted DNA of five resistant strains and one sensitive strain were used as a template, and PCR amplification was carried out by using F/R primers and ITS1/ITS4 primers respectively. The results of agarose gel electrophoresis showed that the fragment sizes of each strain were between 500 and 700 bp (S1 Fig).

#### Comparison of sequences of resistant strains with sensitive strain

The results of PCR amplification of the five resistant strains and one sensitive strain were sent to Shanghai Biotech Co., Ltd. for bidirectional sequencing. Comparison with sensitive strain sequences revealed that the base sequences of propiconazole, hexaconazole, and myclobutanil resistant strains did not change while the partial base pairs of the flusilazole and tebuconazole resistant strains were mutated. The results showed that the base mutation rate of the tebuconazole and the flusilazole resistant strains was 4.8% and 1.6% respectively (S2 and S3 Figs).

## Discussion

The hull-less pumpkin powdery mildew is an obligate parasite, meaning that the pathogen must be stored, cultured and reproduced in the living plant, and it is therefore susceptible to factors such as host growth conditions during reproduction. In 2018, in the first report of powdery mildew caused by *P*. *xanthii* on *Lagenaria siceraria* in China, disease incidence was 85% from a sampled population of 100 plants. Disease severity on individual plants ranged between 10 and 30% [22]. In addition, *P*. *xanthii* could infect watermelon (*Citrullus lanatus*) and chayote (*Sechium edule)* [11,23–28].

### Development of resistance in hull-less pumpkin powdery mildew

Presently, the domestic registered fungicides for the control of cucurbit powdery mildew mainly include triazoles, strobilurins and succinate dehydrogenase inhibitors in sterol biosynthesis inhibitors. Cucurbit powdery mildew has a high prevalence, a high reproduction rate of *P*. *xanthii*, and a large number of reproductive *P*. *xanthii*. Therefore, *P*. *xanthii* is often resistant, and the resistance of *P*. *xanthii* develops rapidly when the fungicide is used continuously [29]. According to the report in 2002 [30], strobilurins, when used alone on a seven-day schedule (usage pattern not mentioned) did not effectively control cucurbit powdery mildew. Strobilurin efficacy declined dramatically after a second application in New York. Efficacy also was reduced in commercial fields in Kentucky and research fields in Arizona, California, Kentucky, Illinois, Michigan, and Virginia in 2002 where strobilurins were used predominantly or exclusively [30]. In the growing seasons of 2012 and 2013, significant declines in cyflufenamid efficacy were observed in two experimental fields in the Apulia (AP) and Emilia-Romagna (ER) regions of Italy on *Cucumis* melo and *C*. *pepo*, respectively. Additionally, EC_50_ values of AP isolates from 2012 and ER isolates from 2013 were greater than those of sensitive isolates, indicating a shift in sensitivity toward resistance to cyflufenamid (resistance factor >100) [31]. In this experiment, when the sensitive strain of *P*. *xanthii* was induced seven generations by using five kinds of triazole fungicide, the sensitive strain becomes the resistant strains and the EC_50_ value of flusilazole, tebuconazole, myclobutanil, and hexaconazole resistant strains were 56.71, 64.62, 47.89, 49.04 times more than that of sensitive strains, which belonged to a higher resistance level. The EC_50_ value of the propiconazole-resistant strain was 19.11 times greater than that of sensitive strains, which showed a moderate resistance level. In Australia, the use of azole fungicides has contributed to the 50-fold increase in canola production in the last 25 years. However, its extensive application sets the stage for the selection of fungal populations with resistance [32]. *Leptosphaeria maculans* was identified with decreased sensitivity to the fungicide fluquinconazole when applied on canola fields as a protective seed dressing. Our study also found that the sensitivity of powdery mildew decreased gradually under the pressure of triazole fungicides selection with the increase of fungicides used times, while its resistance tended to be stable when fungicide use was stopped. In Gansu Province, triazole fungicides such as hexaconazole are widely used for the prevention and control of powdery mildew in hull-less pumpkin. With increasing usage in the field, the control efficacy gradually declined when used four times in a growing season, with some differences in susceptibility to hexaconazole among 20 powdery mildew strains. The EC_50_ value was in the range of 17.77–285.54 μg·mL^-1^, and the resistance factor was 8.94, which was a low resistance [19]. Powdery mildew strain collected from the field were found to display tebuconazole resistance, in order to more effectively control powdery mildew in hull-less pumpkin, it is suggested to rotate or alternate the use of triazole fungicides with a different mechanism.

### Molecular mechanism of resistance to triazole fungicides in hull-less pumpkin powdery mildew

The specific primers F/R were used to amplify the DNA of the resistant strains after continuous induced domestication of five kinds of triazole fungicides in the laboratory for the seventh generation. The DNA sequencing results were compared with the sensitive strain of *P*. *xanthii*, and it was found that some bases points of the resistant strains to tebuconazole and flusilazole had mutated, with mutation rates of 4.80% and 1.60%, respectively. Mutations were not found in the bases of the other three resistant strains. Although the resistance levels of the five resistant strains are relatively stable, the base mutation rate of the resistant strains is closely related to the level of resistance. The resistance factor of tebuconazole is 7.91 times higher than that of flusilazole, and the base mutation rate is increased by 66.67%, it can be seen that the higher the resistance level, the more likely the base is to mutation. According to another report, the mechanisms that drive azole resistance in agriculture were point mutations in the *CYP51* amino acid sequence, overexpression of the *CYP51* enzyme and overexpression of genes encoding efflux pump proteins [33]. Wang li studied the resistance mutation sites of wheat powdery mildew against triazolone and found that the cumulative frequency of resistance mutations at the 136^th^ and 398^th^ sites of the amino acids accounted for 78.78% [18]. These two mutation sites are considered the main sites governing resistance in powdery mildew populations [18]. We have studied only the mutation of the base of the resistant strain in this experiment, and it could not show that the resistance mechanism of resistant strains of *P*. *xanthii* was the same as that of resistant strains of wheat powdery mildew, which was a mutation of a certain site or multiple sites of the *CYP51* gene [17,18].

With the widespread use of triazole fungicides in the prevention and control of *Cucurbita* powdery mildew, the problem of resistance to powdery mildew has become increasingly prominent, which has become the key limiting factor in the effective control of the disease [9]. The fungicide resistance mechanism is of great significance for guiding the effective use of triazole fungicides to control pumpkin powdery mildew. Simultaneously, it lays a theoretical foundation for the development of multi-target sites with different mechanisms of fungicides to control *P*. *xanthii*. However, this experiment only studied the mechanism of resistance to triazoles in ITS region DNA sequences of hull-less pumpkin powdery mildew under laboratory conditions. The resistance mechanism related to the resistance gene *CYP51* and the use of triazole fungicides in the field requires further study.

## Conclusions

The sequence of the resistant strains of *P*. *xanthii* was compared with the sequence of the sensitive strain, and it was found that the base of the resistant strains to tebuconazole and the flusilazole had mutated, and the mutation rates were 4.8% and 1.6% respectively after undergoing triazole fungicide treatment and induction for seven generations. The research results are of great significance for guiding the use of these five triazole fungicides to effectively control pumpkin powdery mildew and delay the development of *P*. *xanthii* resistance.

## Supporting information

S1 Fig The result of PCR amplification of resistance strains of *Podosphaera xanthii*. A: sensitive stain; B: Resistance stain of propiconazole; C: Resistance stain of flusilazole; D: Resistance stain of hexaconazole; E: Resistance stain of myclobutanil; F: Resistance stain of tebuconzole.
(TIF)

S2 Fig Sequence alignment between sensitive strain and tebuconazole-resistance strain of *P*. *xanthii*. (TIF)

S3 Fig Sequence alignment between sensitive strain and flusilazole -resistance strain of *P*. *xanthii*. (TIF)

## References

[1] Radocaj O, Dimic E, Diosady LL, Vujasinovic V. Use of Hull-less pumpkin (*Cucurbita pepo L*.) seed oil press-cake in gluten-free cookies: Nutritional and mineral profile. Agro Food Industry Hi-Tech, 2017; 28(2): 63–66. https://147.91.169.5/handle/123456789/3743

[2] Mohammad HB, Zoleikha A, Arash Z, Sara D, Seyed MM, Bahman JK. Anti-proliferative and apoptotic effects of hull-less pumpkin extract on human papillary thyroid carcinoma cell line. Anatomy & cell biology, 2021; 54(1): 104–111. doi: 10.5115/ACB.20.228 33504684 PMC8017459

[3] Musa S, Nurhan U, Önder T, Fahad AJ, Mehmet MÖ. Chemical compositions and mineral contents of some hulless pumpkin seed and oils. Journal of the American Oil Chemists’ Society, 2015; 93(8): 1095–1099.

[4] Tańska Magorzata, Ogrodowska D, Bartoszewski G, Korzeniewska A, Konopka I. Seed lipid composition of new hybrids of styrian oil pumpkin grown in Poland. Agronomy, 2020; 10(8): 1104. doi: 10.3390/agronomy10081104

[5] Kami D, Ito K, Sugiyama K, Muro T, Morishita M, Noguchi Y. A new pumpkin cultivar “Stripe pepo” with hull-less seed and short internodes. Acta Horticulturae, 2016; 1127: 415–419. doi: 10.17660/ActaHortic.2016.1127.64

[6] Wang HW, Xu YQ. Research progress in the functional factors of pumpkin. Food & Machiney, 2004; 20(4): 55–57. https://kns.cnki.net/kcms/detail/detail.aspx?FileName=SPJX200404022&DbName=CJFQ2004.

[7] Statistics on the total import and export volume and amount of China’s white melon seeds (12129993) from 2014 to 2018. https://www.chyxx.com.

[8] Wang M. Cucurbita—the most Diversity. Chinese watermelon melon, 2002; (3): 42–45.

[9] Liu LL, Liang QL, Zhang AQ, Zhang H. Sensitivity of hull-less pumpkin powdery mildew to five kinds of triazole fungicides. Journal Yangzhou University (Agricultural and life science edition), 2019; 40(2): 101–106. https://kns.cnki.net/kcms/detail/detail.aspx?FileName=JSNX201902016&DbName=CJFQ2019.

[10] Liang QL, Wei LX, Xu BL, Liu LL, Calderón-Urrea A. First Report of Powdery Mildew Caused by *Podosphaera xanthii* on Hull-less Cucurbita pepo of Western District in China. Plant Disease. 2021; 105 (2). doi: 10.1094/PDIS-04-20-0703-PDN 32967556

[11] Lebeda Aleš, Křístková Eva, Sedláková Božena, McCreight James D., Coffey Michael D. Cucurbit powdery mildews: methodology for objective determination and denomination of races. European Journal of Plant Pathology, 2015; 144: 399–410. doi: 10.1007/s10658-015-0776-7

[12] Braun U, Shin HD, Takamatsu S, Meeboon J, Kiss L, Lebeda A, et al. Phylogeny and taxonomy of *Golovino-myces* orontii revisited. Mycological Progress, 2019; 18(3): 335–357. doi: 10.1007/s11557-018-1453-y

[13] Liang QL, Xu BL, Yan HX, Chen RX, Xue YY. Hull-less pumpkin powdery mildew and its host range. Mycosystema, 2010; 29(5): 636–643. https://kns.cnki.net/kcms/detail/detail.aspx?FileName=JWXT201005005&DbName=CJFQ2010.

[14] Li FK, Li P, Li XY, Xue YY. Control Effect of Chinese Medicinal Herb Extracts on Powdery Mildew of Hull-less Pumpkin. China Vegetables, 2020; (3): 56–60. https://kns.cnki.net/kcms/detail/detail.aspx?FileName=ZGSC202003015&DbName=CJFQ2020.

[15] Vanden BH, Marichal P, Odds FC. Molecular mechanisms of drug resistance in fungi. Trends in Microbiology, 1994; 2(10): 393–400. doi: 10.1016/0966-842x(94)90618-1 7850208

[16] Délye C, Laigret F, Corio-Costet M F. A mutation in the 14α-demethylase gene of *Uncinula necator* that correlates with resistance to a sterol biosynthesis inhibitor. Appl Environ Microbiol, 1997; 63: 2966–2970. doi: 10.1128/aem.63.8.2966-2970.1997 9251183 PMC168594

[17] Délye C, Bousset L, Corio-Costet MF. PCR cloning and detection of point mutations in the eburicol 14α-demethylase (CYP 51) gene from *Erysiphe graminis* f. sp. hordei, a “recalcitrant” fungus. Curr Genet, 1998; 34: 399–403. doi: 10.1007/s002940050413 9871123

[18] Wang L. The sensitivity of population of *Blumeria graminis* f.sp. tritici isolates to triadimefon and azoxystrobin and the mutation detection of resistant genes with molecular technology in China. Northwest Agriculture & Forestry University, 2011; p.22-31. https://kns.cnki.net/kcms/detail/detail.aspx?FileName=1011402839.nh&DbName=CMFD2012.

[19] Liang QL, Wei LX, Liu LL, Wu Q, Xu BL. Resistance induction of Podosphaera xanthii in pumpkin to hexaconazole and biological characterization of its resistant strains in Gansu. Plant Preotection. 2018; 44(1): 87–94. https://kns.cnki.net/kcms/detail/detail.aspx?FileName=ZNTB201225026&DbName=CJFQ2012.

[20] Yang P. The sensitivity of population of *blumeria graminis* f. sp tritici isolates to tradimefon and quinoxyfen in China. Northwest Agriculture & Forestry University, 2010; p.23-28. https://kns.cnki.net/kcms/detail/detail.aspx?FileName=2010149968.nh&DbName=CMFD2010.

[21] Chi WJ. Genetic diversity and molecular detection for *Blumiria gramini* sf. sp. *tritici* in Northerneastern China. Shenyang: Shenyang Agricultural University, 2009. https://kns.cnki.net/kcms/detail/detail.aspx?FileName=2009190478.nh&DbName=CDFD2009.

[22] Cui H, Wu C, Zhu Q, Fan C, Gao P, Luan F. First report of powdery mildew caused by *Podosphaera xanthii* on *Lagenaria siceraria* in China. Plant Disease, 2018; 102, (11): 12–17. doi: 10.1094/PDIS-12-17-1993-PDN 30673457

[23] Kousik CS, Donahoo RS, Webster CG, Turechek WW, Adkins ST, Roberts PD. Outbreak of cucurbit powdery mildew on watermelon fruit caused by *Podosphaera xanthii* in Southwest Florida. Plant Disease, 2011; 95(12): 1586–1586. doi: 10.1094/PDIS-06-11-0521 30732000

[24] Singh R, Ferrin DM, Aime MC. First Report of Powdery Mildew Caused by *Podosphaera xanthii* on *Sechium edule* in the United States. Plant Disease, 2009; 93(12): 1348–1348. doi: 10.1094/PDIS-93-12-1348A 30759520

[25] Choi IY, Choi YJ, Shin HD. First report of powdery mildew caused by *Podosphaera xanthii* on *Cucurbita maxima* in Korea. Journal of Plant Pathology, 2020; 102(2): 599–599. doi: 10.1007/s42161-019-00482-5

[26] Cui H, Wu C, Zhu Q, Fan C, Gao P, Luan F. First report of powdery mildew caused by *Podosphaera xanthii* on *Lagenaria siceraria* in China. Plant Disease, 2018. doi: 10.1094/PDIS-12-17-1993-PDN 30192178

[27] Liu XB, Zhang JH, Cui CS. Identification of pathogen and evaluation the resistance to pumpkin powdery mildew in Heilongjiang Province. China Watermelon and Muskmelon. 2006; 1: 10–13. https://kns.cnki.net/kcms/detail/detail.aspx?FileName=EGYP200909001039&DbName=CPFD2010.

[28] Choi IY, Cheong SS, Joa JH, Cho SE, Shin HD. First report of powdery mildew caused by *Podosphaera xanthii* on *Sechium edule* in Korea. Plant Disease. 2015; 99(1): 162–162. doi: 10.1094/PDIS-10-14-1011-PDN 30699759

[29] Han HH, Ma T, Xie B. Progress on the resistance inducing to *Cucurbits* powdery mildew and its mechanism. Chinese Agricultural Science Bulletin. 2012; 28(25): 124–128.

[30] Mcgrath MT, Shishkoff N. First Report of the Cucurbit powdery mildew fungus (*Podosphaera xanthii*) resistant to strobilurin fungicides in the United States. Plant Disease. 2003; 87(8): 1007–1007. doi: 10.1094/PDIS.2003.87.8.1007A 30812786

[31] Pirondi A, Nanni IM, Brunelli A, Collina M. First report of resistance to cyflufenamid in *Podosphaera xanthii*, causal agent of powdery mildew, from melon and zucchini fields in Italy. Plant Disease, 2014; 98(11): 1581–1581. doi: 10.1094/PDIS-02-14-0210-PDN 30699832

[32] Angela P. Van de Wouw, Vicki LE, Steven C, Francisco J, López-Ruiz Steven J, et al. Identification of isolates of the plant pathogen *Leptosphaeria maculans* with resistance to the triazole fungicide fluquinconazole using a novel *In Planta* assay. PLoS ONE. 2017;12(11): 1–19. doi: 10.1371/journal.pone.0188106 29141039 PMC5687775

[33] Parker JE, Warrilow A, Price CL, Mullins J, Kelly SL. Resistance to antifungals that target CYP51. Journal of Chemical Biology. 2014; 7(4): 143–161. doi: 10.1007/s12154-014-0121-1 25320648 PMC4182338

