# Supplementary figures and images for: Induction of resistance of Podosphaera xanthii (hull-less pumpkin powdery mildew) to triazole fungicides and its resistance mechanism
Source: PLoS One. 2022 Feb 1;17(2):e0263068. doi: 10.1371/journal.pone.0263068 (PMC8806069; doi:10.1371/journal.pone.0263068)

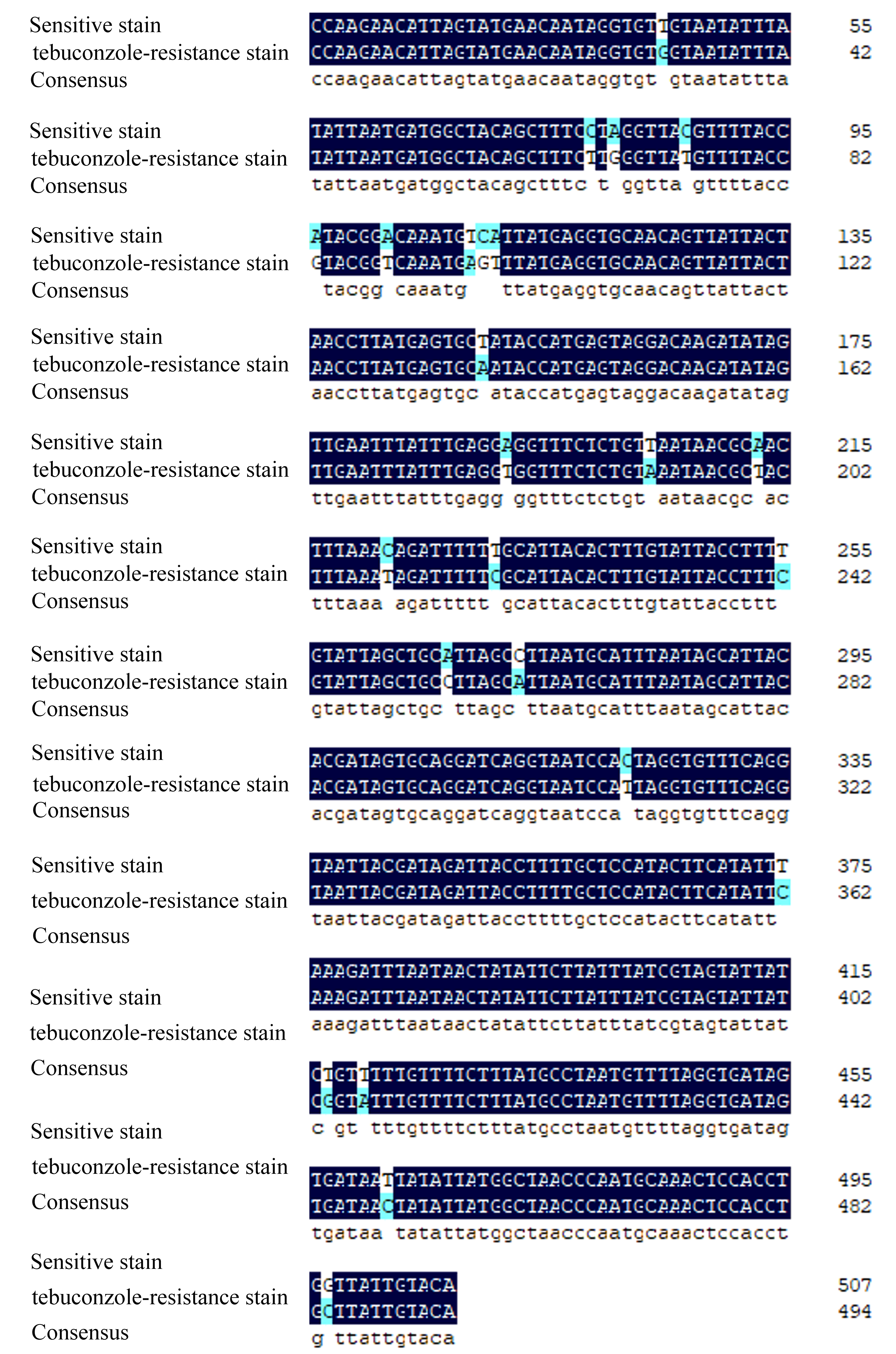

Supplement: S2 Fig — (TIF) [file pone.0263068.s002.tif]

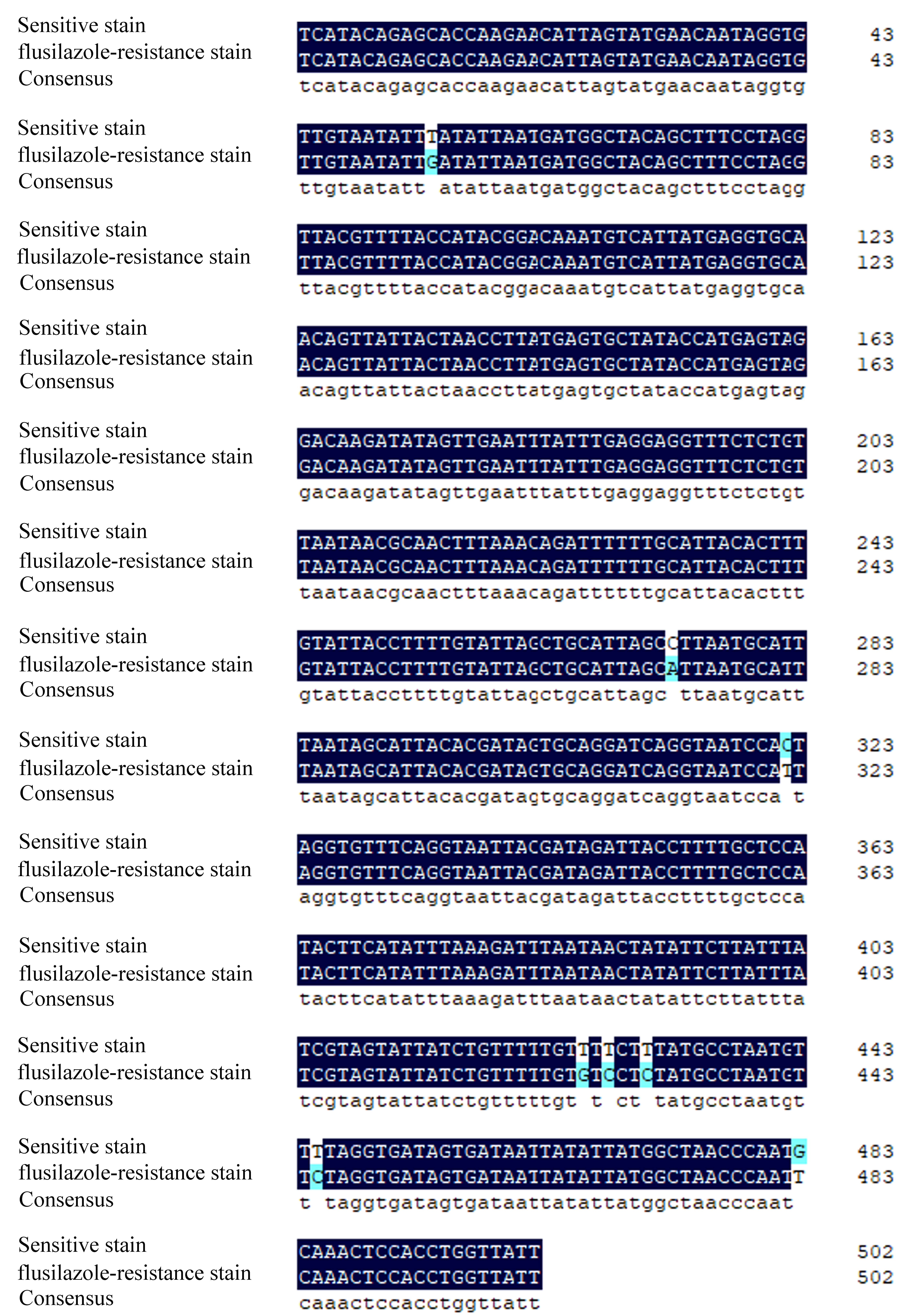

Supplement: S3 Fig — (TIF) [file pone.0263068.s003.tif]
